# Unraveling transcriptomic signatures and dysregulated pathways in systemic lupus erythematosus across disease states

**DOI:** 10.1186/s13075-024-03327-4

**Published:** 2024-05-13

**Authors:** Frank Qingyun Wang, Li Shao, Xiao Dang, Yong-Fei Wang, Shuxiong Chen, Zhongyi Liu, Yujing Mao, Yuping Jiang, Fei Hou, Xianghua Guo, Jian Li, Lili Zhang, Yuting Sang, Xuan Zhao, Ruirui Ma, Kai Zhang, Yanfang Zhang, Jing Yang, Xiwu Wen, Jiong Liu, Wei Wei, Chuanpeng Zhang, Weiyang Li, Xiao Qin, Yao Lei, Hong Feng, Xingtian Yang, Chun Hing She, Caicai Zhang, Huidong Su, Xinxin Chen, Jing Yang, Yu Lung Lau, Qingjun Wu, Bo Ban, Qin Song, Wanling Yang

**Affiliations:** 1 Department of Paediatrics and Adolescent Medicine, The University of Hong Kong, Hong Kong, China; 2 Department of Rheumatology and Lupus Research Institute, Affiliated Hospital of Jining Medical University, Jining, Shandong China; 3 School of Life and Health Sciences, School of Medicine, and Warshel Institute for Computational Biology, The Chinese University of Hong Kong - Shenzhen, Shenzhen, Guangdong China; 4 Medical Research Center, Affiliated Hospital of Jining Medical University, Jining, Shandong China; 5 Department of Endocrinology, Affiliated Hospital of Jining Medical University, Jining, Shandong China; 6 Department of Paediatrics, Faculty of Medicine, The Chinese University of Hong Kong, Hong Kong, China; 7 Medical Laboratory of Jining Medical University, Jining Medical University, Jining, Shandong China; 8 Department of Rheumatology and Clinical Immunology, Chinese Academy of Medical Sciences & Peking Union Medical College, Beijing, China

**Keywords:** Systemic lupus erythematosus, Remission, PBMC, Gene signatures

## Abstract

**Objectives:**

This study aims to elucidate the transcriptomic signatures and dysregulated pathways in patients with Systemic Lupus Erythematosus (SLE), with a particular focus on those persisting during disease remission.

**Methods:**

We conducted bulk RNA-sequencing of peripheral blood mononuclear cells (PBMCs) from a well-defined cohort comprising 26 remission patients meeting the Low Lupus Disease Activity State (LLDAS) criteria, 76 patients experiencing disease flares, and 15 healthy controls. To elucidate immune signature changes associated with varying disease states, we performed extensive analyses, including the identification of differentially expressed genes and pathways, as well as the construction of protein-protein interaction networks.

**Results:**

Several transcriptomic features recovered during remission compared to the active disease state, including down-regulation of plasma and cell cycle signatures, as well as up-regulation of lymphocytes. However, specific innate immune response signatures, such as the interferon (IFN) signature, and gene modules involved in chromatin structure modification, persisted across different disease states. Drug repurposing analysis revealed certain drug classes that can target these persistent signatures, potentially preventing disease relapse.

**Conclusion:**

Our comprehensive transcriptomic study revealed gene expression signatures for SLE in both active and remission states. The discovery of gene expression modules persisting in the remission stage may shed light on the underlying mechanisms of vulnerability to relapse in these patients, providing valuable insights for their treatment.

**Supplementary Information:**

The online version contains supplementary material available at 10.1186/s13075-024-03327-4.

## Introduction

Systemic Lupus Erythematosus (SLE) is characterized by variable clinical manifestations and an unpredictable disease course [1–4]. Disease severity is measured using the Systemic Lupus Erythematosus Disease Activity Index 2000 (SLEDAI) score, with higher scores indicating more severe conditions [5]. The treatment goal is to achieve complete remission (SLEDAI = 0) or Low Lupus Disease Activity State (LLDAS) (SLEDAI ≤ 4), as patients experiencing longer periods in these states demonstrate improved clinical outcomes [6, 7]. While over 50% of SLE patients achieve remission through immunosuppressant treatments, permanent remission remains uncommon, and many patients experience flare-ups [8–11]. As such, understanding the molecular signature at various time points during the disease course is essential for developing personalized and effective treatments for different stages of SLE.

Transcriptomic studies focusing on RNA expression levels have identified several molecular signatures crucial to SLE. Previous research has demonstrated that immune response pathways, including type I interferon, plasmablast, and neutrophil, are significantly upregulated in SLE patients, while lymphoid cell signatures, such as T cells, B cells, and NK cells, are downregulated [12, 13]. The plasmablast signature has been found to best predict disease activity, and neutrophil signatures are associated with lupus nephritis [14, 15]. Recently, researchers have employed advanced single-cell RNA-seq technology to investigate changes at the cellular and molecular level, uncovering multiple disease-specific cell subtypes that could potentially play a pathogenic role in SLE development [16, 17]. However, most studies have focused on transcriptomic changes between SLE patients and healthy controls, neglecting the disease's heterogeneity within its course. Patients achieving stable remission may exhibit a distinct transcriptomic profile compared to those actively experiencing flares.

In a previous study, we observed that intensive in-hospital treatment increased the neutrophil signature while mitigating the interferon (IFN) signature in SLE patients [18]. In the current study, we focused on SLE patients in remission and aimed to use bulk RNA-seq technology to examine the transcriptomic profile of peripheral blood mononuclear cells (PBMCs) from various SLE patients, including those in the acute phase requiring hospitalization, those with consistently low disease activity, and healthy controls. Through differential gene expression and functional enrichment analysis, we identified specific signatures that were alleviated during remission, potentially serving as markers to track disease activities. Moreover, our analysis uncovered signatures persisting in patients in remission, which could be potential treatment targets for preventing disease relapse.

## Patients and methods

### Study design and samples collection

In a previous study, we compared transcriptomic profiles of SLE patients before and after intensive in-hospital treatment [18]. In the current study, we recruited additional patients from Jining Medical University Affiliated Hospital in China, resulting in a cohort of 15 healthy controls, 76 active patients requiring immediate in-hospital treatment, and 26 remission patients. All patients were diagnosed based on the American College of Rheumatology revised criteria [19], and SLEDAI scores were measured using SLEDAI-2K [5]. Remission patient samples were collected during medical follow-up, with all patients meeting the LLDAS criteria for at least one month, having a SLEDAI score ≤ 4, and a current prednisolone (or equivalent) dose ≤7*.*5*mg* [20]. Fourteen of the 26 remission patients were among those followed up longitudinally, who had blood samples collected during the active disease state, after intensive in-hospital treatment and during remission. IRB approval and written informed consent were obtained from all participants. The clinical characteristics of the patients are summarized in Table 1.

**Table 1:** Clinical characteristics of the study participants

Feature	SLE active patients (*n* = 76)	SLE remission patients (*n* = 26)
Age	37 [24.25,49.5]	38.5 [31.5,48]
Female Sex (%)	65 (85)	23(88)
SLEDAI	10.5 [9, 14]	2 [0,2]
Clinical Parameter		
Anti-dsDNA	410.4 [162.1,800]	2.64 [2.07.7.67]
IgG	16.7 [12.15,20.7]	13.95 [12.5,16.43]
Lymphocyte Count	0.92 [0.6575,1.375]	1.915 [1.415,2.217]
White Blood Cell Count	3.935 [2.915,6.673]	5.865 [5.082, 7.590]
C3	0.59 [0.36, 0.85]	0.93 [0.84, 1.07]
C4	0.09 [0.04, 0.16]	0.19 [0.15, 0.255]
Treament (%)		
Prednisolone	76 (100)	26 (100)
Hydroxychloroquine	68 (89)	21 (81)
DMARDs	9 (12)	0 (0)
MMF	12 (16)	9 (35)
Cyclophosphamide	12 (16)	0 (0)
Rituximab	4 (5)	0 (0)

*DMARD* Disease-modifying antirheumatic drugs, *MMF* Mycophenolate mofetil

^*^Except where indicated otherwise, values are Interquartile Range (IQR)

### RNA sequencing and data processing

Ten milliliters of blood were collected from both SLE patients and healthy donors using heparin tubes. PBMCs were isolated from the samples via Ficoll-Paque PLUS centrifugation, following standard protocols (Ficoll-Paque™, Cytiva). The samples were stored in -80°C after lysis with TRizol (TRIzol™ Reagent, Thermo Fisher). Total RNA was extracted and quality checked by agarose gel electrophoresis, Nanodrop, Qubit and Agilent 2100. The NEBNext® Ultra™ RNA Library Prep Kit was employed for RNA library preparation, and samples were sequenced using paired-end 150 bp reads on an Illumina platform at Novogene, Beijing, China. To ensure data quality, raw RNA sequencing data underwent quality control using Fastp [21]. Reads were mapped to the hg38 human genome from Gencode V44 utilizing the STAR alignment algorithm [22], and gene-level read counts were quantified using featureCounts [23].

### Analysis of differential gene expression and functional analysis

DESeq2 was used to identify differentially expressed genes (DEGs), applying a threshold of adjusted *p*-value < 0.05 and log2 fold change > 0.5 or log2 fold change < -0.5 [24]. Gene expression data were normalized using the Variance Stabilizing Transformation (VST) method from DESeq2. Functional enrichment analysis was conducted using the R package clusterProfiler, incorporating information from Gene Ontology (GO), Kyoto Encyclopedia of Genes and Genomes (KEGG), and Reactome terms [25]. Moreover, Gene Set Variation Analysis (GSVA) was performed on the VST-normalized gene expression data, using a manually curated list of immune-related genes extracted from previous studies [26–29]. This analysis calculates sample-wise gene set enrichment scores [30]. Differentially expressed gene sets based on the GSVA scores were identified by the R package limma, defined as adjusted *p*-values < 0.01 and log2 fold change > 0.6 [31].

### Cell deconvolution analysis

We utilized CIBERSORTx for cell deconvolution analysis to infer the relative abundance of immune cells from the gene expression profile in PBMCs, based on the previously established Leukocyte signature matrix (LM22) comprising 547 genes [32]. By using VST-normalized gene expression data as input, this analysis enabled us to compare the relative abundance of various cell types among healthy controls, patients with active disease, and patients in remission.

### Cell type enrichment analysis

We conducted a Cell Type Enrichment Analysis (CSEA) to identify specific cell types potentially enriched in different disease states, using the list of DEGs identified in our study. In brief, CSEA employs a permutation-based test for cell-type specificity, which leverages the gene expression patterns within a comprehensive single-cell RNA sequencing dataset that covers 186 lymphatic system cell types [33]. This analysis enabled us to pinpoint the cell types that may be enriched in various disease stages based on their associated DEGs.

### Protein-protein interaction network analysis

To construct the protein-protein interaction network, we imported the genes into the Search Tool for the Retrieval of Interacting Genes (STRING), which infers interactions based on evidence from high-throughput experiments, databases on Protein-Protein interactions (PPI), co-expression of the relevant genes, and shared function in the same metabolic pathways, with a minimum requirement score indicating the confidence of interaction set to 0.4 [34]. We visualized the resulting networks using Cytoscape and identified functional modules using the Molecular Complex Detection (MCODE) plugin. A score greater than 10 was considered a significant core module, and other parameters were set as follows: degree cutoff = 2, node score cutoff = 0.2, K-score = 2, and max. depth = 100. These parameters generally measure the centrality and connectivity of the nodes in the network.

### Connectivity map drug repurposing analysis

We performed a drug repurposing analysis using the L1000 assays in the Connectivity Map (cMAP) database through the clue.io interface, which records the in vitro effects of perturbagens on gene expression in each of the nine cell lines studied [35]. We imported genes from the functional modules identified in the Protein-protein interaction network analysis described earlier into the cMAP platform. By querying these genes, cMAP generated enrichment scores that reflect the relationship between the input gene signature and the gene expression reference profiles for the drugs tested. A more negative enrichment score (ES *<* -80) indicates that a drug may have reversed the expression pattern of the genes in a given gene list, making it a potential treatment candidate for the disease state characterized by the expression profile of the identified module.

### Statistical analysis

All statistical analyses were conducted in R. We calculated pathway scores for GSVA terms and PPI-defined functional modules by applying a previously described method to the VST-normalized gene expression [36]. We correlated pathway scores with clinical features using a linear regression model implemented in the R function lm(). To determine the differences in functional module scores among various patient groups, we utilized the Wilcoxon signed-rank test and paired t-test implemented in the ggpubr package in R.

## Results

### Transcriptomic profiling of SLE patients

**Fig. 1:**
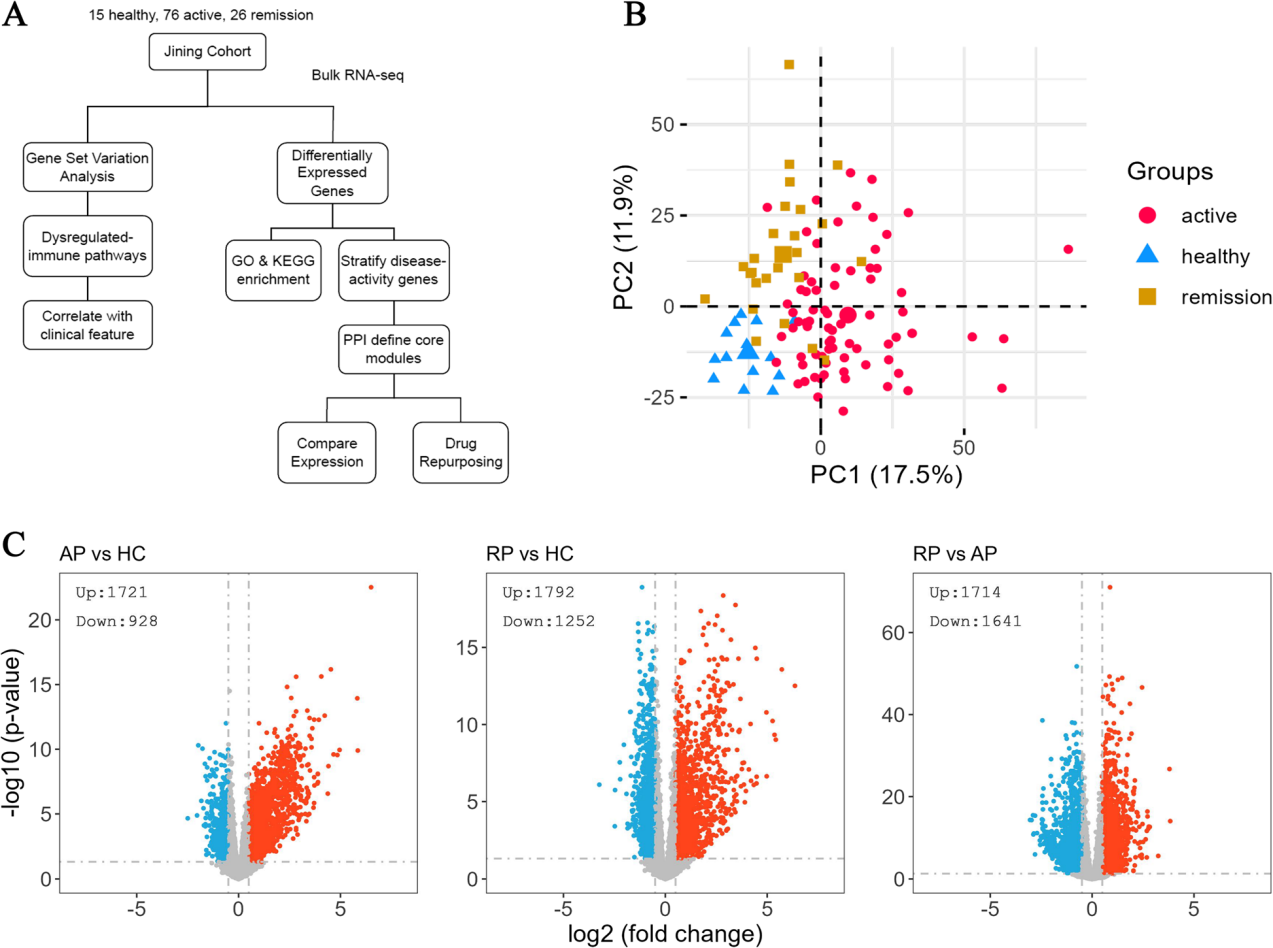
Transcriptomic profiling of SLE patients. **A** Study design and workflow of the study. **B** Principal component analysis. **C** Volcano plot of the differentially expressed genes in Active Patients vs Healthy Controls (left), Remission Patients vs Healthy Controls (middle), Remission Patients vs Active Patients (right). (AP: Active Patient, RP: Remission Patient, HC: Healthy Control)

The Principle Component Analysis (PCA) revealed a distinct but not fully separated relationship between remission patients and active patients (variance explained by PC1:17.5%, PC2:11.9%). Both patients in active and remission states demonstrated significantly higher heterogeneity compared to the healthy control group. This emphasizes the intricate nature of SLE in all phases of the disease (Fig. 1B).

We utilized DESeq2 to conduct the analysis of DEGs, applying a threshold of p.adjust *<* 0.05 and log2Fold change *>* 0.5. This resulted in over 5000 distinct genes in the three comparisons. Specifically, we identified 2,649 genes in Active patients vs Healthy controls (1,721 up, 928 down), 3,044 genes in Remission patients vs Healthy controls (1,792 up, 1,252 down), and 3,355 genes in Remission patients vs Active patients (1,714 up, 1,641 down) (Fig. 1C). Detailed DESeq2 results can be found in Supplementary Table 1-3.

**Fig. 2:**
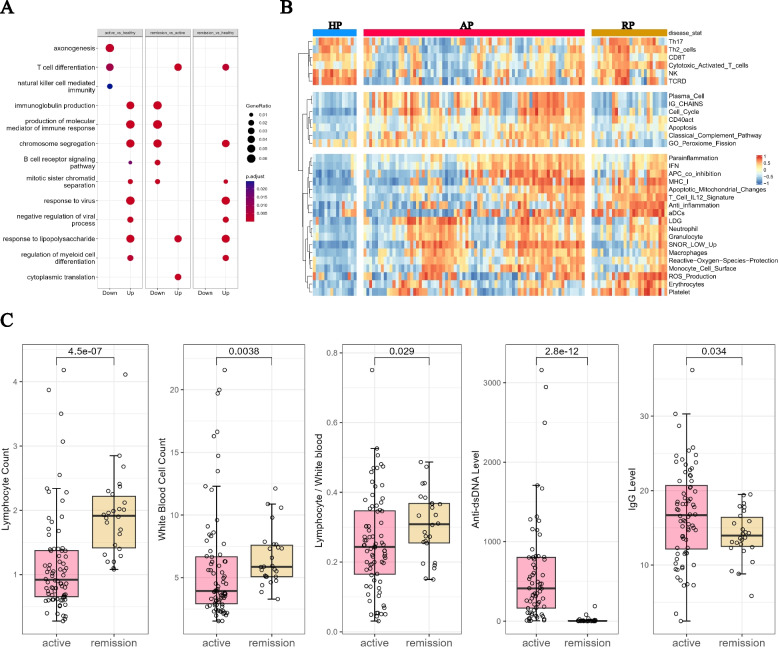
Molecular signatures in the SLE patients in different disease states. **A** GO enrichment analysis of the DEGs among the three groups. **B** Heatmap of the differentially expressed immune-related pathways identified from GSVA analysis. The top panel contains the pathways that are down-regulated in active patients and recovered in remission patients. The middle panel contains the pathways that are up-regulated in active patients and down-regulated in remission patients. The bottom panel contains the pathways that are up-regulated in active patients and remained up-regulated in remission patients (AP: Active Patient, RP: Remission Patient, HC: Healthy Control). **C** Boxplot of the clinical records compared between active patients and remission patients

We performed functional enrichment analysis on the differentially expressed genes (Fig. 2A). We found that terms such as “immunoglobulin production” and “production of molecular mediator of immune response” were enriched with up-regulated genes in active patients compared to controls. However, these terms returned to normal expression levels in patients under remission. On the other hand, the term “T cell differentiation” was down-regulated in active patients but recovered in patients with remission. Additionally, terms such as “response to virus”, “response to lipopolysaccharide”, and “regulation of myeloid cell differentiation” were up-regulated in both patients with flare and remission compared to controls (Fig. 2A).

### Pathway signatures

To gain a deeper understanding of the molecular characteristics associated with different disease states, we conducted GSVA using a meticulously curated list of immune gene sets based on several previous studies [26–29]. Pathway scores were calculated for each sample and collectively compared across the three disease states. Our findings revealed that T-cell related pathways, such as “Th17” and “NK” were significantly downregulated in active patients but restored in remission patients (Fig. 2B). Further analysis using cell type deconvolution showed a similar pattern for resting NK cells, which was likely due to lymphopenia in active SLE patients that improves during remission (Supplementary Fig 1C). This observation aligns well with the clinical records, which demonstrated an increase in lymphocyte/white blood cell counts in patients during remission compared to active patients (Fig. 2C).

Conversely, pathways such as “IG CHAINS”, “Plasma Cell”, and “Cell Cycle” were significantly upregulated in active patients but recovered during remission, indicating their close association with disease activity (Fig. 2B). This finding is consistent with clinical records showing that remission patients generally exhibit lower anti-dsDNA and IgG levels compared to active patients (Fig. 2C). Similarly, the results of cell deconvolution analysis revealed an increase in the percentage of plasma cells in active patients and back to normal level in remission patients (Supplementary Fig 1C).

Additionally, several pathways, including “IFN”, “MHC-I”, “Neutrophil”, and “ROS Production” were consistently upregulated in both remission and active patients compared to healthy controls (Fig. 2B). These pathways serve as persistent disease signatures throughout different disease states.

### Correlation between transcriptomic signature and clinical manifestations

**Fig. 3:**
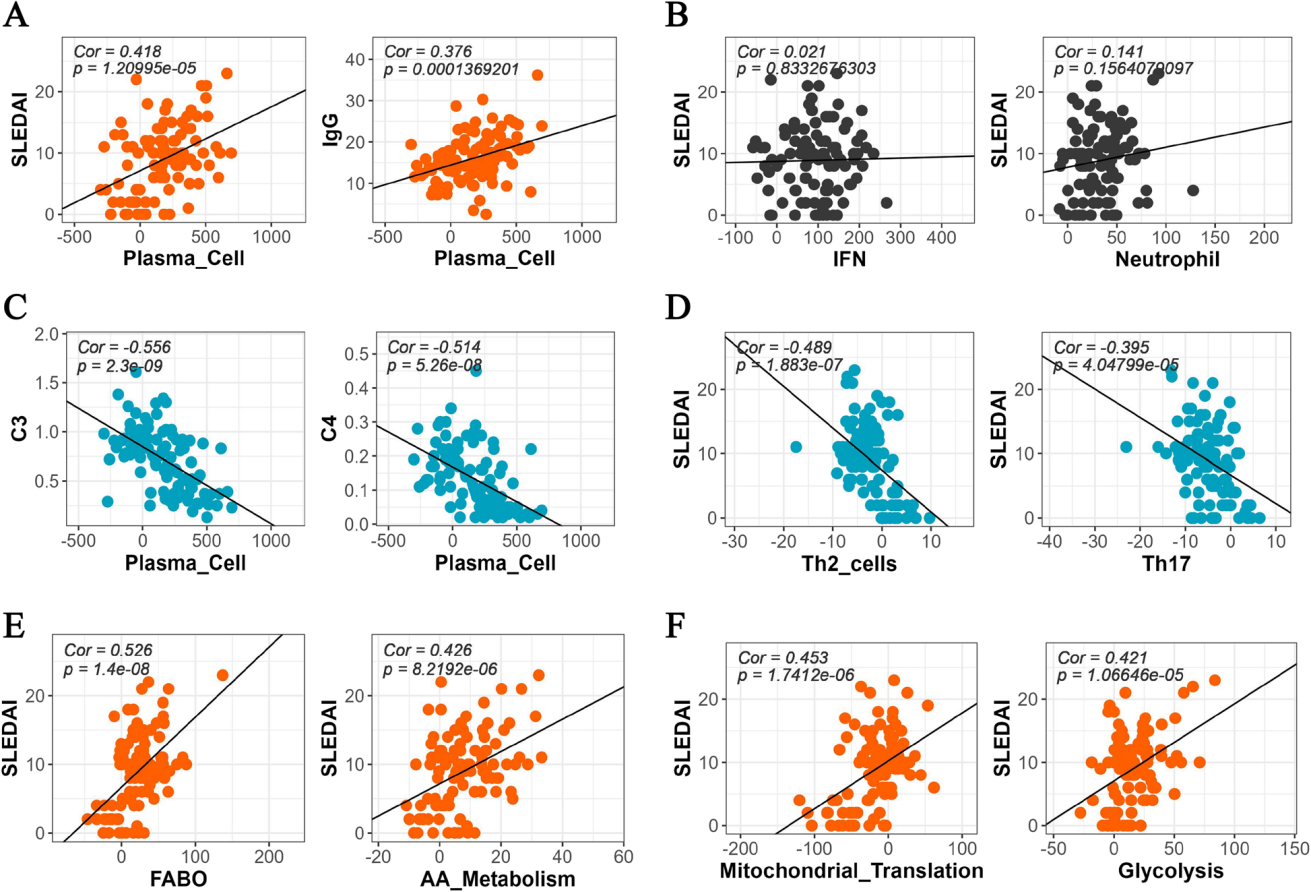
Correlation between transcriptomic signatures and clinical features with Pearson coefficient and *p*-value shown. **A** Positive correlation of plasma cell signature with SLEDAI score and IgG level. **B** No significant correlation observed between IFN/Neutrophil signatures and SLEDAI score. **C** Negative correlation of plasma cell signature with C3 and C4 level. **D** Negative correlation of Th2/Th17 signatures with SLEDAI score. **E**-**F** Positive correlation of fatty acid beta oxidation, amino acid metabolism, mitochondrial translation, glycolysis signatures with SLEDAI score

We further investigated the relationship between transcriptional signatures and clinical manifestations by conducting a correlation analysis of pathway scores and clinical features with a correlation coefficient *>* 0.35 and *p* value *<* 0.05 set as cut-off. Our findings revealed positive correlations between immune pathways such as “Plasma Cell” and both the SLEDAI score and IgG level (Fig. 3A). Additionally, several metabolism-related pathways, including “Fatty Acid Beta Oxidation”, “Amino Acid Metabolism”, and “Mitochondrial Translation”, were also significantly correlated with the SLEDAI score, underscoring the involvement of immunometabolism in autoimmunity (Fig. 3E-F).

Moreover, the “Plasma Cell” signature demonstrated a strong inverse relationship with C3 and C4 levels, corroborating the use of transcriptomic signatures to track clinical features (Fig. 3C). In contrast, the “Th2” and “Th17” pathways displayed negative correlations with the SLEDAI score (Fig. 3D). Notably, we observed no correlation between the “IFN” and “Neutrophil” pathways and the SLEDAI score, which may be attributed to their persistent nature throughout the disease course (Fig. 3B).

### Network analysis revealed core modules involved in the course of the disease

**Fig. 4:**
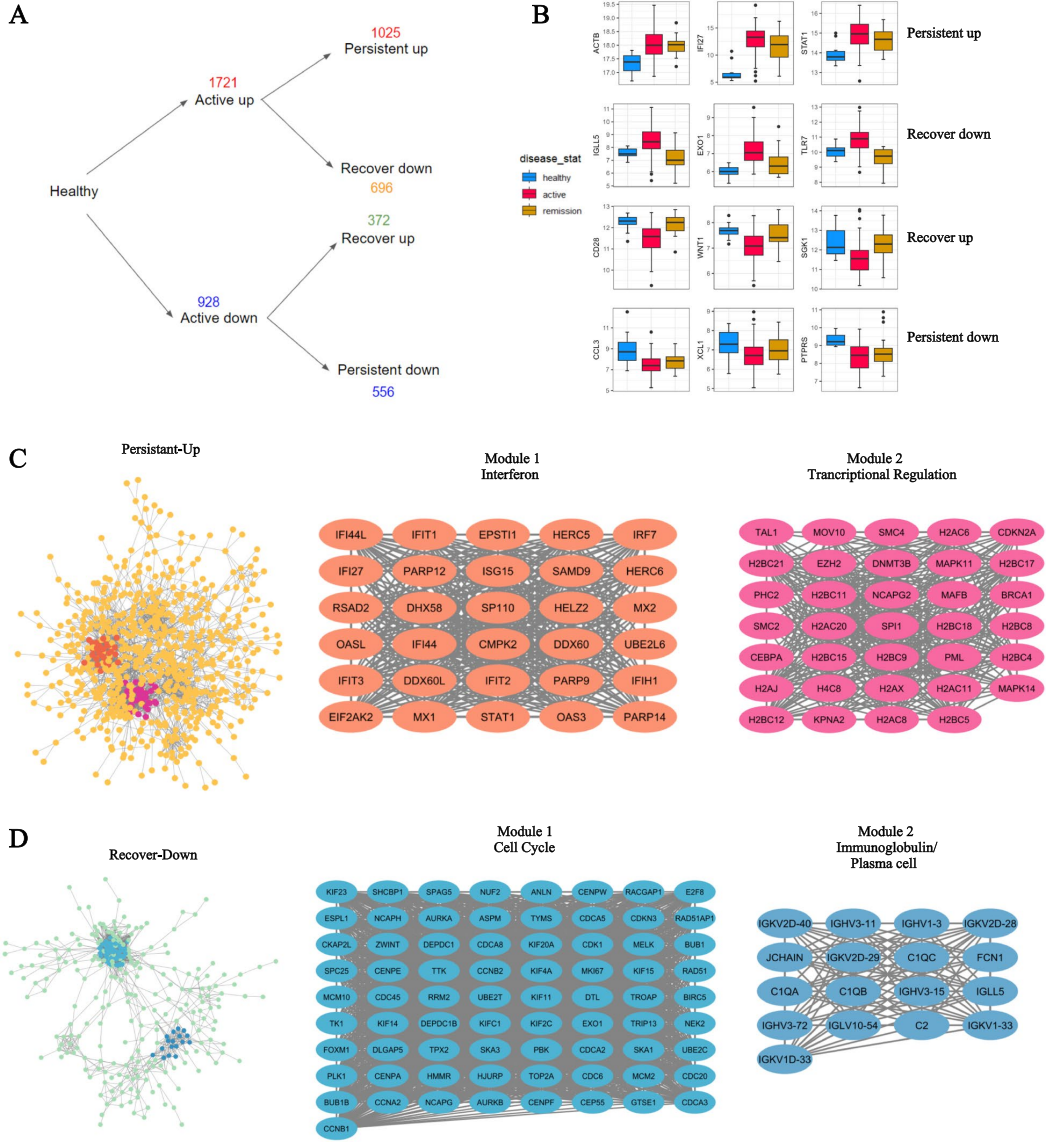
Stratification of the genes dysregulated in active SLE patients. **A**-**B** Overview and examples of genes altered in active SLE patients. There are 1721 genes up-regulated in the active patients, of which 696 significantly decreased in remission patients and 1025 remained unchanged. There were 928 genes down-regulated in the active patients, of which 372 significantly increased in remission patients and 556 remained unchanged (**C**) Protein-Protein interaction network and the identification of core modules of the genes persistently up-regulated in the remission patients compared to the healthy control. **D** Protein-Protein interaction network and the core modules of the genes down-regulated in the remission patients comparing to the active patients

To categorize dysregulated genes in active patients, we stratified them based on their expression changes during active disease and in remission. For genes that were upregulated in active patients compared to healthy individuals, those with a reduced expression of 0.5 log2fold and an adjusted *p *value less than 0.05 in remission patients compared to active patients were classified as “Recovered Down”. Genes that did not meet this criterion were classified as “Persistent Up”. A similar approach was used to classify “Persistent Down” and “Recovered Up” genes (Fig. 4A).

Examples of “Persistent Up” genes include STAT1, ACTB, and IFI27, while genes classified as “Recovered Down” include IGLL, EXO1, and TLR7 (Fig. 4B). The CSEA analysis reveals that persistent up genes are enriched in myeloid lineage cells, including monocytes, macrophages, and neutrophils. In contrast, the Recovered Down genes are primarily significantly enriched in plasma cells (Supplementary Fig 2A-B).

We built Protein-Protein Interaction Networks for genes classified as “Persistent Up” and “Recovered Down” to examine the complex interactions between the genes in each category. In order to pinpoint genes with essential functional connections in each network, we employed the MCODE algorithm, which identified core modules with scores exceeding 10. Gene Ontology and Reactome pathway enrichment analyses were conducted to generate functional annotations for these modules.

Among the genes classified as “Persistent Up”, we observed two modules. Module 1 exhibited enrichment in terms such as “GO: defense response to virus”, “GO: response to external biotic stimulus”, and “Reactome: Interferon Signaling”. Upon querying the Interferome database (http://interferome.org), we found that all these genes are core interferon-regulated genes shared among Type I, Type II, and Type III mechanisms (Supplementary Fig 1D). Genes in Module 2 demonstrated enrichment in terms such as “Reactome: Oxidative Stress Induced Senescence” and “Reactome: Transcriptional regulation of granulopoiesis” (Fig. 4C).

Regarding the “Recovered Down” genes, Module 1 displayed enrichment in terms such as “Reactome: Cell Cycle” and “GO: cell cycle process”. On the other hand, Module 2 exhibited enrichment in terms such as “GO: immunoglobulin complex” and “Reactome: Initial triggering of complement” (Fig. 4D).

### Expression change of core modules throughout the disease course

**Fig. 5:**
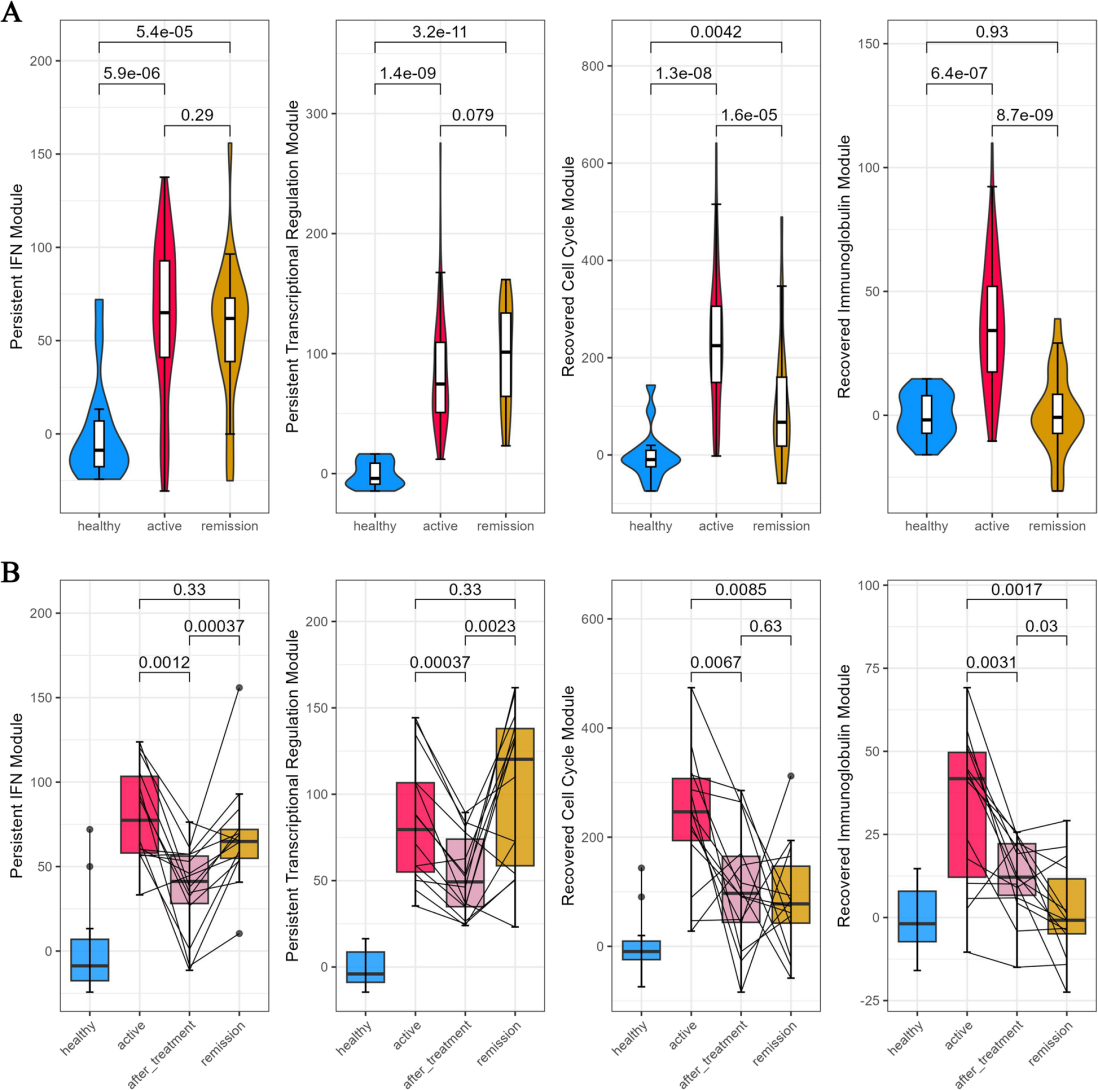
Expression level of the functional modules identified from PPI analysis throughout the disease course. **A** Violin plot shows the expression level of persistent IFN module, persistent transcriptional regulation module, recovered-down cell cycle module, recovered-down immunoglobulin module among healthy, active patients and remission patients. **B** Boxplot illustrates the expression changes of the functional modules in paired samples among different disease stages such as active disease, after intensive hospital treatment, and during remission

We then evaluated the expression level of the functional modules throughout the disease course. In both active and remission patients, the interferon and transcriptional regulation modules sustained a higher level of expression than healthy controls (Fig. 5A). By leveraging the samples who have paired data across the active state, immediately after intensive in-hospital treatment and the remission period, we observed that the IFN and transcriptional regulation modules were repressed to a lower level after intensive in-hospital treatment but bounced up during the remission period (Fig. 5B). In contrast, in the remission patients, the cell cycle and immunoglobulin modules significantly decreased compared to the active patients (Fig. 5A). Particularly, the cell cycle module decreased but to a level that is still significantly higher than healthy controls after intensive in-hospital treatment and throughout the remission. The decreased immunoglobulin module after intensive treatment kept decreasing and returned to the level of healthy controls in patients under remission (Fig. 5B).

### Potential compounds to reverse persistent-up module expression

**Fig. 6:**
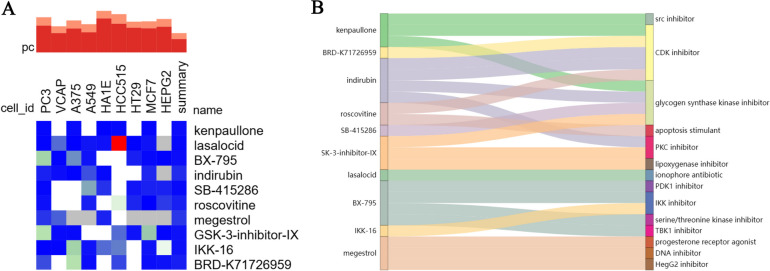
Connectivity Map Drug Repurposing Analysis. **A** Heatmap of the enrichment score of the top 10 perturbagens in 9 cell lines. **B** Sankey diagram of the top 10 molecules predicted to reverse the expression of the genes in the persistent up core modules with their mechanisms of actions

After importing the genes from the two persistent-up modules to the cMAP database, we identified potential small molecule compounds capable of reversing their expression signature. A more negative ES score indicates that the compounds are more capable of reversing the expression of the given gene set. The top molecules with the most negative mean score across the nine cell lines include kenpaullone, palbociclib, SB-415286, BX-795, IKK-16, GSK-3-inhibitor-IX, and AT-9283, which are classified as CDK inhibitors, IKK inhibitors, and Glycogen Synthase Kinase inhibitors (Fig. 6A-B). Then, we imported the genes from the two Recovered-Down modules to the cMAP. Interestingly, we identified widely-used SLE drugs, including mycophenolate mofetil, mycophenolic-acid, methotrexate, and corticosteroid as top perturbagens to reverse the expression of the recovered genes (Supplementary Table 6). The concordance with the clinical drug usage showcases the potential utility of drug repurposing based on in-vitro transcriptomic changes.

## Discussion

This study focused on analyzing the transcriptomic profile of well-defined SLE remission patients who met the LLDAS criteria for a certain period, confirmed through medical follow-up. The unpredictable nature of SLE remission and relapse cycles makes it challenging for patients to manage their symptoms and adhere to treatment plans, leading to increased healthcare costs and reduced treatment effectiveness [3]. Low-dose immunosuppressants typically stabilize patients for only a limited time, with a large proportion of patients experiencing flares afterward [11]. The discovery of molecular biomarkers can help distinguish between active and inactive disease states, monitor disease flares, and predict the likelihood of future flares, allowing for more personalized and effective treatment plans. We performed an extensive functional analysis aimed at characterizing the specific features of SLE remission state.

Our analysis demonstrated that the most significant distinguishing features between remission patients and active patients were the up-regulated lymphocyte signatures, as well as the down-regulated plasma and cell cycle signatures. Lymphopenia is a prevalent condition in SLE, characterized by a decrease in the number of lymphocytes in the blood [37, 38]. As revealed by GSVA and cell deconvolution analysis, the decline of T and NK cell activities observed in active SLE patients may indicate a state of lymphopenia, which has been discussed in previous transcriptomic research [13]. Furthermore, our study showed that these lymphocyte signatures recovered in remission patients and had a negative correlation with the SLEDAI score, suggesting a restoration of lymphopenia conditions during remission.

Plasma cells play a significant role in the pathogenesis of SLE. Specific subsets of plasma cells, such as plasmablasts and long-lived plasma cells, can be found in the circulation and affected tissues of SLE patients. These cells not only produce autoantibodies but also secrete pro-inflammatory cytokines and chemokines, perpetuating the autoimmune response and amplifying inflammation in SLE [39–41]. Previous studies have demonstrated a positive relationship between plasma signatures and disease activity in SLE [13]. In line with these findings, our analysis further validated a positive association between the plasma cell and SLEDAI score, indicating that increased plasma cell activity played a key role to heightened disease activity. Additionally, we observed a negative correlation between the plasma cell/Ig chain signature and C3/C4 levels, which is consistent with decreased complement levels often observed in active SLE patients [42]. By utilizing GSVA analysis and examining the expression of immunoglobulin modules identified through PPI, we determined that plasma cell signatures largely returned to normal levels during remission.

Additionally, we identified a downregulation of a core signature associated with cell cycle function in patients under remission compared to active patients. This finding aligns with previous research showing enrichment of disease-activity genes in cell cycle pathways, supporting the notion that dysregulated cell cycle processes contribute to SLE pathogenesis [43, 44]. Although the expression levels of the genes in cell cycle module in remission patients were lower than in the active state, they remained higher than in healthy individuals. This highlights the potential importance of restoring proper cell cycle regulation in achieving complete remission.

Upon comparing SLE patients in both flare and remission to healthy controls, we observed upregulated GO terms such as "response to virus" and "regulation of myeloid cell differentiation", indicating a deregulated innate immune response throughout the course of the disease. Similarly, GSVA analysis showed that several innate immune pathways, including interferon, neutrophil, parainflammation, and ROS production, consistently exhibited up-regulation in patients with remission. Neutrophils contribute to tissue damage and inflammation through the release of reactive oxygen species and pro-inflammatory cytokines [45]. They also form neutrophil extracellular traps, which can promote autoantibody production and immune complex deposition [46]. Dysregulation of the interferon pathway is a characteristic feature of SLE and contributes to the chronic inflammation and autoimmunity observed in the disease [12].

The PPI analysis identified a core Persistent-Up module consisting solely of 30 interferon regulated genes. Another core module identified through PPI analysis consisted of histone genes and transcriptional regulation-related genes, such as MOV10 and SPI1. Dysregulation of these genes suggests an unstable and disturbed genomic architecture in SLE patients, even during the remission state, underlying the risk for further disturbance of gene expression. These pathways have a weak correlation with disease activities, likely due to their high levels of expression even in patients with low disease activity.

The paired data shows that intensive in-hospital treatment with large doses of immunosuppressants temporarily suppressed their expression to a lower level, but they rebounded during remission. This suggests that even for patients in a stable remission state who exhibit none or limited clinical symptoms, there may still be ongoing immune dysregulation. These immune pathways, which fail to be suppressed by current treatments, may pose a risk for chronic damage and potential relapse in SLE patients.

Analyzing the genes from the two Persistent-Up core modules in cMAP, we identified small compound molecules, including IKK inhibitors and CDK inhibitors that could reverse their expression in vitro. Previous research has shown that blocking CDK activity could reduce inflammation activity in a lupus-prone mouse model, while inhibiting NFKB pathways reduced IFN expression in vitro, highlighting their potential role in treating persistent features in patients under remission [47, 48].

In addition to small compound molecules, biologics targeting type I interferons have undergone extensive clinical testing. Anifrolumab, which targets the IFN-alpha receptor, has been proven to reduce disease severity in patients with moderate-to-severe SLE [49]. Together with traditional immunosuppressants, targeting these persistently upregulated genes appears to be a promising therapeutic approach for treating patients with remission and further controlling disease activity.

At the cellular level, the genes that fail to be suppressed are likely preferentially expressed in monocytes. Monocytes play a crucial role in the pathogenesis of SLE. Previous studies have demonstrated that monocytes from SLE patients exhibit dysregulated inflammatory features, such as heightened IFN production and deregulated cell cycle regulation [17, 50]. A distinct subset of monocytes, characterized by the coexpression of IL1B and IFN genes, has been specifically identified in SLE patients [16]. It is likely that current treatment strategies have failed to control the activities of monocytes in SLE remission patients. Thus, understanding the pathogenicity of these dysregulated monocytes is crucial for developing novel therapeutic strategies that address the root cause of the disease.

The current study has several limitations. We could not track the disease activities for all the patients, so we treated the remission and active patients as two separate cohorts with distinct disease states. Additionally, we had a limited number of remission patients that met our selection criteria. Moreover, the bulk level data and the analysis do not directly reveal transcriptomic changes at the cellular level, which could have more accurately reflected changes in cell subpopulations. Future studies should address these issues, possibly by utilizing larger cohorts, conducting long-term longitudinal follow-ups, and employing cutting-edge technologies such as single-cell RNA-seq.

## Conclusion

Overall, our analysis of the transcriptomic profile of SLE remission patients has yielded critical insights into the immune signatures throughout disease course. Our research highlights the distinct features of SLE remission, with a specific emphasis on the recovery of lymphocyte signature and the attenuation of plasma and cell cycle pathways. However, the persistent signatures, including IFN modules and transcriptional regulation modules, contribute to the chronic inflammation observed in patients, even in the absence of clinical symptoms. These findings present a promising avenue for the development of biomarkers to track disease activity in SLE patients. Additionally, our research offers a foundation for the development of personalized treatment strategies that could lead to more effective management of SLE.

### Supplementary Information

**Additional file 1.** 

**Additional file 2: Sup Figure 1.** (A-B) KEGG and Reactome enrichment analysis of the DEGs between three groups. (C) Boxplots show the cell proportion of NK and plasma cells among healthy, active, remission patients. (D) Venn diagram present the classification of the genes in the persistent IFN modules. (E) Boxplots show the SLEDAI score of the patients in active state, after in-hospital treatment, and in remission. 

**Additional file 3: Sup Figure 2.** (A-B) Jitter plot shows the enrichment of persistent up (up panel) and recovered down genes (down panel) in certain cell types. The red dotted line represents the Bonferroni-corrected *p* value threshold.

## Data Availability

The datasets used and/or analysed during the current study are available from the corresponding author on reasonable request.

## Abbreviations

SLE: Systemic lupus erythematosus
SLEDAI: Systemic Lupus Erythematosus Disease Activity Index 2000
LLDAS: Lupus Low Disease Activity State
PBMC: Peripheral Blood Mononuclear Cell
IFN: Interferon
DEG: Differentially Expressed Gene
GSVA: Gene Set Variation Analysis
CSEA: Cell Type Enrichment Analysis
PPI: Protein-Protein Interaction
cMAP: Connectivity Map
VST: Variance Stabilizing Transformation
