# Supplementary figures and images for: Unraveling transcriptomic signatures and dysregulated pathways in systemic lupus erythematosus across disease states
Source: Arthritis Res Ther. 2024 May 13;26:99. doi: 10.1186/s13075-024-03327-4 (PMC11089778; doi:10.1186/s13075-024-03327-4)

A

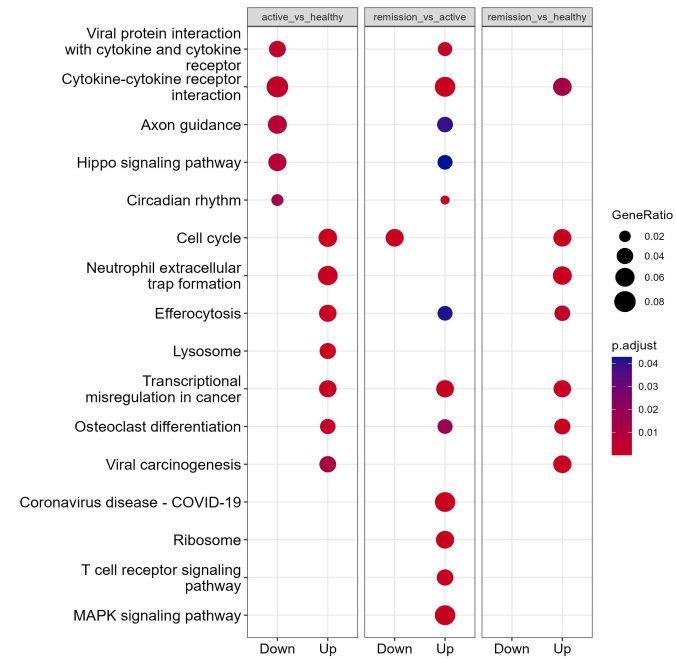

B

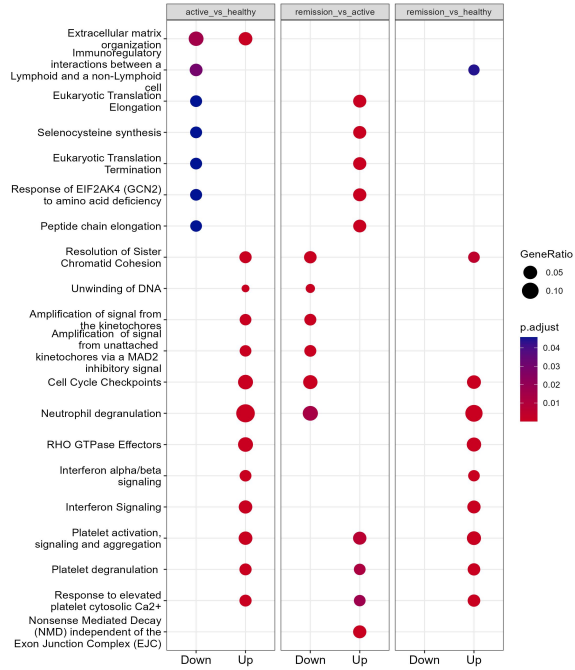

C

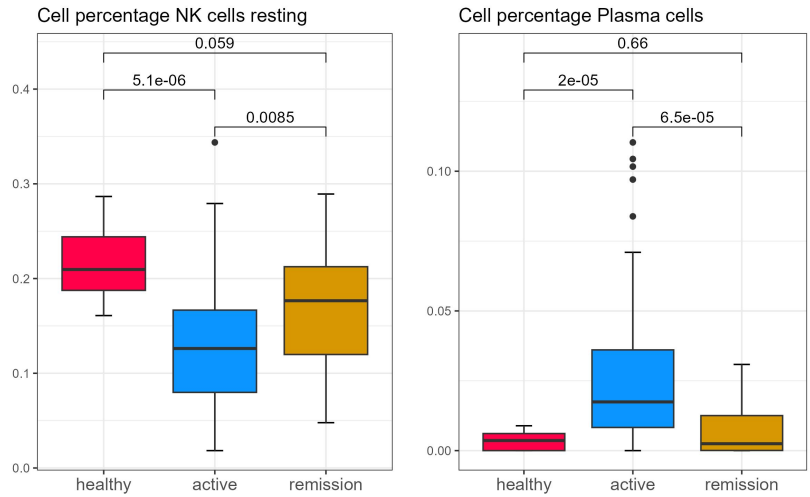

D

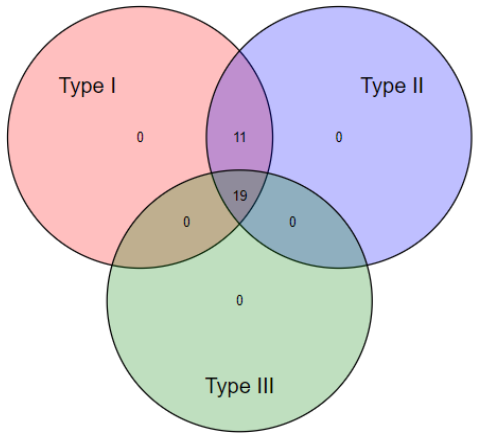

E

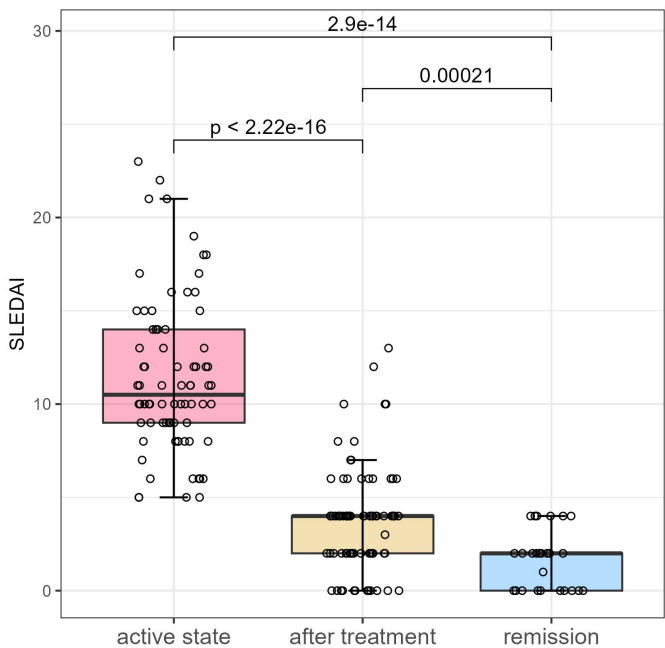

Supplement: Supplementary file 2 — Additional file 2: Sup Figure 1. (A-B) KEGG and Reactome enrichment analysis of the DEGs between three groups. (C) Boxplots show the cell proportion of NK and plasma cells among healthy, active, remission patients. (D) Venn diagram present the classification of the genes in the persistent IFN modules. (E) Boxplots show the SLEDAI score of the patients in active state, after in-hospital treatment, and in remission. [file 13075_2024_3327_MOESM2_ESM.pdf]

**A**

### Top20 general cell types

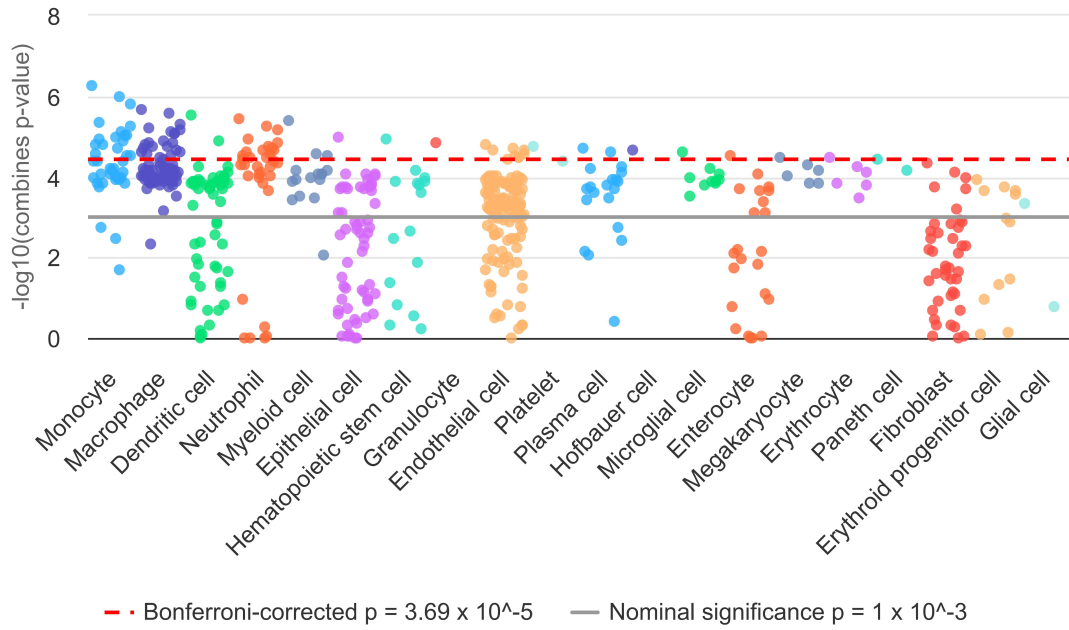

**B**

### Top20 general cell types

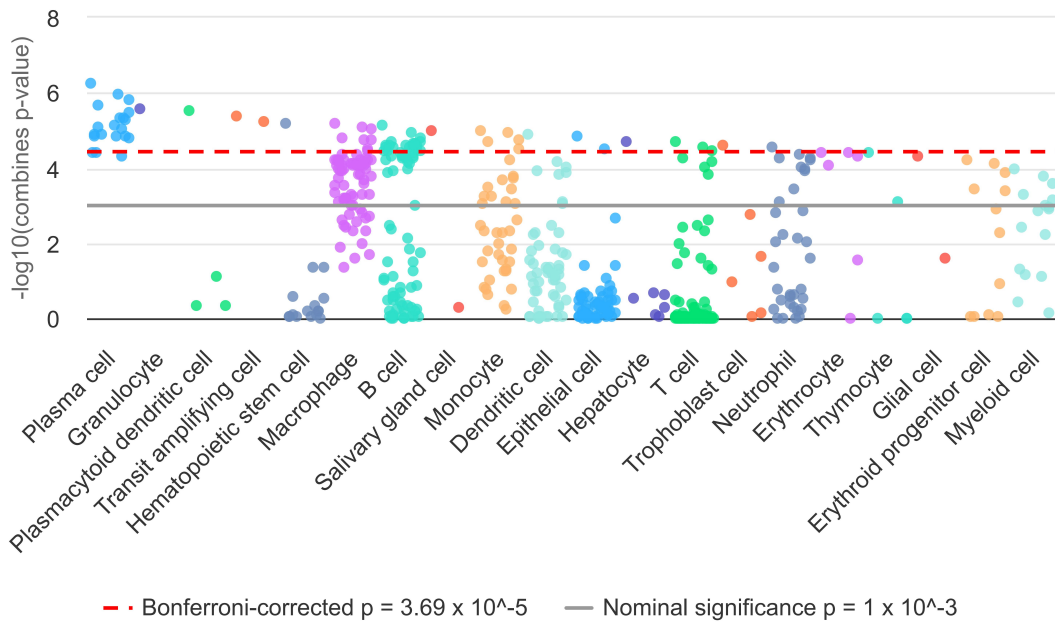

Supplement: Supplementary file 3 — Additional file 3: Sup Figure 2. (A-B) Jitter plot shows the enrichment of persistent up (up panel) and recovered down genes (down panel) in certain cell types. The red dotted line represents the Bonferroni-corrected p value threshold. [file 13075_2024_3327_MOESM3_ESM.pdf]
